# Micronutrient intakes in the Dutch diet: foods, fortified foods and supplements in a cross sectional study

**DOI:** 10.1007/s00394-023-03219-4

**Published:** 2023-08-05

**Authors:** Julia K. Bird, Maaike J. Bruins, Marco E. Turini

**Affiliations:** 1 Bird Scientific Writing, Wassenaar, The Netherlands; 2 dsm-firmenich, Health, Nutrition & Care, Kaiseraugst, Switzerland; 3 Present Address: dsm-firmenich, Taste, Texture & Health, Delft, The Netherlands

**Keywords:** Vitamins, Minerals, Dietary adequacy, Food fortification, Supplements, The Netherlands

## Abstract

**Purpose:**

This study investigates intakes of risk micronutrients from non-fortified foods, fortified foods and food supplements in different age and gender sub-groups of the Dutch population.

**Methods:**

This is a secondary analysis of the Dutch National Food Consumption Survey (DNFCS 2012–2016, *N* = 4313, 1–79 years). The proportion of the population with Habitual Intakes below the Estimated Average Requirement (EAR) and above the Upper Level (UL) for calcium, iron, zinc, vitamin A, vitamin B6, folate, vitamin D and vitamin E from non-fortified foods, fortified foods and total intake including food supplements was calculated using Statistical Program to Assess Dietary Exposure (SPADE).

**Results:**

More than 50% of the population had an intake below the EAR for calcium, iron, vitamin D and folate. Intakes were inadequate for certain sub-groups for the other vitamins and minerals. Adolescents and women were the population sub-groups most likely to have an intake below the EAR. For zinc, vitamin A and folic acid, more than 1% of toddlers exceeded the UL from the total intake. A negligible proportion exceeded the UL for the other vitamins and minerals.

**Conclusion:**

Inadequate intakes were found for several micronutrients in various population sub-groups despite an apparently well-nourished population. Intakes of zinc, folic acid and vitamin A from food supplements in toddlers and preschoolers should be investigated further to ensure they do not exceed recommended amounts. These results can be used to inform policy makers and to design nutritional interventions to improve micronutrient intakes in the Netherlands.

**Supplementary Information:**

The online version contains supplementary material available at 10.1007/s00394-023-03219-4.

## Introduction

Adequate intakes of vitamins and minerals (micronutrients) are important for public health and to avoid deficiency disease in individuals [1]. Low micronutrient status can adversely affect health outside of frank deficiency, for example low vitamin C status causes fatigue and lethargy, causing a negative impact on quality of life and reduced work performance. Sub-clinical deficiency will progress to the disease scurvy if the deficiency is not corrected [2]. Despite economic prosperity, low nutrient intakes are a concern in high income countries [3, 4]. Some population sub-groups remain at increased risk of malnutrition [5]. For example, adolescents have high nutrient needs relative to their energy intake, thus placing them at greater risk of inadequate intakes of nutrients such as protein, vitamin A and calcium. Sub-groups at risk of food insecurity may consume a nutrient-poor diet or have low food intakes, and thus have low overall micronutrient intakes. Furthermore, high-income countries may not consider micronutrient deficiencies as being a concern and there may be a lack of adequate surveillance as other health problems receive more attention.

The legislation surrounding the fortification of foods in the Netherlands is complex. Mandatory fortification is not allowed; however, voluntary fortification between 15 and 100% is allowed for most vitamins and minerals except preformed vitamin A, vitamin D, folic acid, selenium, copper and zinc, for which fortification is permissible for restoration or substitution purposes only. An agreement between the government and industry promotes fortification of margarines with vitamins A and D, and salt with iodine [6]. Food supplements (also referred to as dietary supplements [7]) are concentrated vitamins, minerals and other ingredients in dose form intended to supplement the diet. They are allowed to be sold in the Netherlands according to the European directive on food supplements, which determines minimum and maximum amounts permitted and health claims that are allowed on packaging and in advertisements [8]. More than one third of Dutch adults use food supplements, and use has been increasing over several decades [9].

The Dutch National Food Consumption Survey (DNFCS) conducted by the National Institute for Public Health and the Environment (RIVM) is designed to “provide insights into the amount of food and drink consumed … to achieve healthy, sustainable and safe food, food product innovation, and to conduct research on education and nutrition.” Previous analyses in the Dutch population using this dataset have been published [6, 10, 11]. The main report “The Diet of the Dutch” provides estimations of long-term, average intakes of micronutrients from foods, including fortified foods, and food supplements together; however, the contribution of each source of micronutrients has not been reported [11]. De Jong and co-workers investigated the effects of fortified foods on micronutrient intakes and adequacy in the Netherlands, however did not look at intakes from food supplements [6].

While it is important to avoid micronutrient deficiencies, chronic high intakes can have adverse health effects, and these need to be kept in mind when considering total micronutrient supply to a population. The consumption of micronutrients through food supplements may pose an additional consideration due to their concentrated form. Fortified foods can be beneficial in increasing general micronutrient intakes over the population; however, the risk of inadequate intakes needs to be balanced with excessive intakes [12, 13]. A comparison of several European countries (Denmark, Germany, Finland, Ireland, Italy, the Netherlands, Poland, Spain and the United Kingdom) found that intakes in excess of the UL were found for preformed vitamin A, zinc, iodine, copper and magnesium [3]. The base diet was the major contributor of nutrients, with fortified foods making only a modest contribution. The contribution of food supplements varied considerably between countries [3].

The aims of this analysis therefore are to investigate the effects of micronutrient source (non-fortified foods, fortified foods and food supplements) on long-term average intakes, micronutrient adequacy and risk of exceeding the UL for select vitamins and minerals in the general Dutch population.

## Methods

### Study population and data sources

The study population included non-institutionalized individuals aged > 12 months who provided at least 1 day of intake in the DNFCS 2012–2016, with the exception of pregnant and lactating women, and those without adequate command of the Dutch language [14]. The main aim of DNFCS 2012–2016 is to gain insights into the diet of children and adults aged 1–79 years living in the Netherlands. The survey was conducted for the RIVM and was designed to be representative for age, gender, geographic region, degree of urbanization and education level. The study population was selected by a market research company based on a consumer panel. Complete data were obtained from 4313 adults and children on a rolling basis in the years 2012–2016 [15].

The study was conducted according to the guidelines of the Declaration of Helsinki. The Medical Ethical Committee of the University Medical Centre Utrecht evaluated the study design prior to the start of data collection (reference number 12-359/C) [15]. Data were obtained with permission from the RIVM. The data were anonymized and could not be linked back to participants.

The micronutrients chosen were based on the vitamins and minerals identified by the European Commission Health and Consumer Protection of having a low (folic acid, vitamin D and vitamin E) or high risk (calcium, iron, zinc, preformed vitamin A, and vitamin B6) of exceeding the UL, i.e., having a moderate or small margin between the highest intake (97.5th percentile) from all dietary sources and the UL [16]. These micronutrients were also identified by Food Supplements Europe as having a low or high risk of exceeding the UL as they are close to the 97.5th percentile, and are referred to as Population Safety Index group 3 micronutrients [17]. Therefore, this analysis describes intakes of calcium, iron, zinc, vitamin A (expressed as total Retinol Activity Equivalents (RAE) and preformed vitamin A only for the UL), vitamin B6, folate (expressed as total Dietary Folate Equivalents (DFE), and synthetic folic acid for the UL), vitamin D and vitamin E [18].

### Questionnaires and dietary intake assessment

The methods have been described in detail elsewhere [14, 15]. Briefly, a general questionnaire was given to all participants containing questions about demographics (age, gender), food supplement intake (general intake, intake different types of supplements, and supplement intake frequency), and the season of completion of the questionnaire. Eight age groups for each gender were chosen to match reference dietary intake categories, with an additional split between younger (19–49 years) and older (50–69 years) adults. We refer to children aged 1 year as toddlers, 2–3 years as preschoolers, 4–8 years as early school-aged children, 9–13 years as younger adolescents, 14–18 years as older adolescents, and 70–79 years as elderly participants. Demographics and nutrient intake data were complete for all participants and there was no missing data.

Dietary intake data were based on two non-consecutive 24-h dietary recalls conducted with an interval of approximately four weeks, using a computer-directed interview program GloboDiet^©^ provided by the International Agency for Research on Cancer (IARC, Lyon, France). All subjects had two dietary recall days. The foods described were matched to the national food composition database (Nederlands Voedingsstoffenbestand, NEVO) and the FoodEx2 classification and description system [15]. Both individual food intake datasets (i.e., a list of all food items consumed per individual), and nutrient intake datasets summarized by day per individual were used in this analysis. The nutrient intake dataset contains intakes from non-fortified and fortified foods combined. In the dataset, the variable *retinol* included both retinol and retinyl esters (M. H. de Jong, personal communication), and was considered to be total preformed vitamin A. Total RAE were calculated according to the formula preformed vitamin A + β-carotene/6 + α-carotene/12 + β-cryptoxanthin/12, which is the method employed by the NEVO national nutrient database [15]. Accordingly, folate equivalents were calculated as the amount of folate naturally present in foods (in µg) plus 1.7 times the amount of folic acid in enriched foods (in µg) plus 2.0 times the amount of folic acid in food supplements (in µg) [15]. Intakes of micronutrients from food supplements on the two dietary recall days were also recorded.

Foods containing fortified micronutrients were flagged in the food intake dataset. For the micronutrient amounts in the fortified food items, it was not possible to distinguish between intrinsic (naturally occurring) and added/fortified micronutrients especially in foods that have a micronutrient content that depends on the exact recipe used by the manufacturers. Therefore, we took a pragmatic approach and assumed that the total micronutrient content for the fortified nutrient was from fortification. Intakes of fortified micronutrients from individual foods were aggregated per participant and per day. To obtain the intake of micronutrients from non-fortified foods, hereafter referred to as the base diet, daily intakes of fortified foods for each day were subtracted from the combined non-fortified and fortified micronutrient intakes.

The DNFCS 2012–2016 of the RIVM provided data on food supplement intake frequency per season (winter, outside winter) and per food supplement class based on a questionnaire [15]. For each individual, the highest frequency of intake of food supplements containing each of the micronutrients of interest for the season in which the survey was taken was used as the supplement frequency (Online resource: Supplemental Tables 1 and 2). Potential food supplement use categories were also created for each micronutrient: Food supplement users of each micronutrient were identified by either a non-null intake frequency for the nutrient from the questionnaire, or non-null intake of each micronutrient from the dietary recall. Zero intake of nutrients from food supplements was considered non-use. Non-users were thus participants reporting no food supplement use and zero intake of each micronutrient from food supplements.

### Habitual Intake analysis

Several methods are available to calculate the long-term average intake of foods and nutrients in populations [19]. The Statistical Program to Assess habitual Dietary Exposure (SPADE), a program in R developed by the RIVM, is one of the methods described. To differentiate the estimates of long-term average intakes by SPADE from those produced by other methods, we refer to Habitual Intakes further in this article.

SPADE was used to calculate Habitual Intake of micronutrients from the base diet (non-fortified foods), base and fortified diet (non-fortified and fortified foods together), and from their total intake (non-fortified foods, fortified foods, and food supplements). Intakes from base and base + fortified diets were calculated with the 1-part model. A 3-part model was used for intakes from the total intake, to account for the differences in model parameters and intake distribution between intakes from food and food supplements. For the 3-part model, micronutrient intakes from the base + fortified diet were estimated for supplement non-users (part 1) and supplement users (part 2), and Habitual Intake from food supplements was performed separately due to the spiked distribution of food supplement intakes (part 3). Population Habitual Intake was obtained by combining total intakes from supplement non-users and users, in a “first-shrink-then-add” approach [20]. The resulting Habitual Intake distribution modeled as a function of age was used to estimate the proportion of subjects below the EAR or AI and above the UL. Dutch dietary guidelines were used as they were considered to be most appropriate for the Dutch population. Cut-off points were used for each age and gender category from the Dutch dietary guidelines: For calcium, vitamin D, vitamin E and folate, different age groups were given either an AI or EAR [1]. Because EAR values were only available for adults and not children, they were estimated from the adult Population Reference Values (PRV) by dividing by the calculated adult two times the standard deviation for males, females, or both genders (ranging from 1.2–1.4 except iron: 1.8–2.1). The Dutch dietary intake guidelines have been harmonized with the EFSA guidelines; however, there are several differences [1, 21]. The AI for vitamin D is higher in the EFSA DRVs (15 mcg) than in the Netherlands (10 mcg). The EAR for vitamin B6 is higher in the EFSA DRVs (1.5 mg for males/1.3 mg for females) compared to in the Netherlands (1.1 mg). Folate requirements for adults are higher in the EFSA DRVs (200 mcg) compared to the Netherlands (200 mcg). For zinc intakes, EFSA specifies a higher zinc DRV when the diet is high in phytates (up to 10.2–12.7 mg), compared to the Netherlands (6.4 mg for males, 5.7 mg for females). The European Food Safety Authority (EFSA) ULs were used if Dutch guidelines were not available [22]. Confidence intervals were quantified via bootstrapping [20].

For this analysis, the SPADE program (R package SPADE.RIVM version 4.1.00 with RStudio 2022.07.0 build 548) was run using both base and base + fortified food intakes, including bootstrapping to estimate confidence intervals. Program defaults were used, including the use of 100 pseudo persons and 200 bootstrap samples, 95% confidence interval for the bootstrap procedures, with a seed of 10. The weighting variable available in the DNFCS “w_demog_season_wk_wknd” was used to account for demographics, season, and survey day of the week. Within SPADE, modeling of micronutrient intakes from food for non-supplement and supplement users was based on age using a fractional polynomial, with no intake frequency set. Intake amounts from food supplements were modeled by age, with intake frequencies modeled according to age with a logistic beta-binomial model per the user’s manual [23]. Due to > 10% zero intakes on dietary recall days for synthetic folic acid from fortified foods, a 2-part model was used with intake frequencies modeled using a cubic spline for this nutrient. The SPADE outputs used in this publication were the median (P50) intake, percent below the EAR/AI, percent above the UL and 95% confidence intervals (lower and upper bound), for females and males (for each age group).

### Other statistical analyses and data visualization

Table 1 was produced using package pollster (0.1.4). Statistical tests between food supplement users and non-users in Supplemental Table 3 and 4 were performed with package TableOne (version 0.13.2) using the default hypothesis tests, which are a chi-square test for categorical variables and a regular ANOVA for continuous variables. Figures were produced using packages ggplot2 (version 3.3.5) and cowplot (version 1.1.1).

**Table 1:** Fortified food and food supplement users by age category

Gender	Age category (years)	*N*^a^	Fortified food user (%)	Fortified food non-user (%)	Standard error fortified food (%) *	Supplement user (%)	Supplement non-user (%)	Standard error food supplements (%) *
Boys	1–3	332	96.2	3.81	2.79	74.4	25.6	6.36
Girls	1–3	340	95.5	4.49	2.98	73.2	26.8	6.37
Boys	4–8	261	95.5	4.51	3.41	50.5	49.5	8.21
Girls	4–8	259	97.8	2.25	2.44	56.5	43.5	8.18
Boys	9–13	259	93.6	6.38	4.03	36.7	63.3	7.95
Girls	9–13	260	91.0	9.02	4.71	44.6	55.4	8.18
Boys	4–18	270	87.8	12.2	5.29	29.8	70.2	7.39
Girls	4–18	254	88.4	11.6	5.34	37.8	62.2	8.07
Men	19–30	260	85.4	14.7	5.82	33.4	66.6	7.76
Women	19–30	256	78.9	21.1	6.77	44.0	56.0	8.23
Men	31–50	259	75.1	24.9	7.13	32.9	67.1	7.75
Women	31–50	264	71.7	28.3	7.35	53.8	46.2	8.14
Men	51–70	264	68.9	31.1	7.56	29.8	70.2	7.47
Women	51–70	258	68.5	31.5	7.67	58.7	41.3	8.14
Men	71–79	260	69.0	31.0	7.61	35.5	64.5	7.88
Women	71–79	257	68.4	31.6	7.70	52.5	47.5	8.27

Weighted for socio-demographic factors, season and day of the week

^*^95% CI

^a^Unweighted Ns are reported

## Results

This dataset from the DNFCS 2012–2016 has been described in detail in other publications [6, 14]. Demographic variables and information about survey seasons are provided in Online Resource: Supplemental Table 3 as unweighted data. Table 1 contains information about fortified food consumers and food supplement users within population age and gender groups. The most frequently consumed food supplements per age group are listed in Online Resource: Supplemental Table 5. Fortified food use decreased as age categories increased, and there was little difference between males and females. The most common types of fortified foods and the micronutrients used as fortificants is listed in Online Resource: Supplemental Table 6. Supplement use was highest in toddlers and young children, was low in older children, adolescents and adults, and increased again in the elderly age category. Adult women were more likely than men to use food supplements.

### General trends in the habitual intake of micronutrients

Intake distributions for all micronutrients for the base diet, fortified food, and food supplements for all age and gender groups are provided in the file Online Resource: Intake Distributions. Habitual Intakes of micronutrients tend to be lower in women than men and increase with age until the elderly age cohort is reached and intakes reach a plateau. Although fortified foods increase micronutrient intakes, their contribution to reducing the proportion below the EAR/AI is modest in general. The effect on total intakes from food supplements was moderate but they reduced the proportion not meeting the EAR to a greater extent than fortified foods. Habitual Intakes for toddlers, preschoolers and elderly participants had wider confidence intervals than other age groups.

### Calcium

**Fig. 1:**
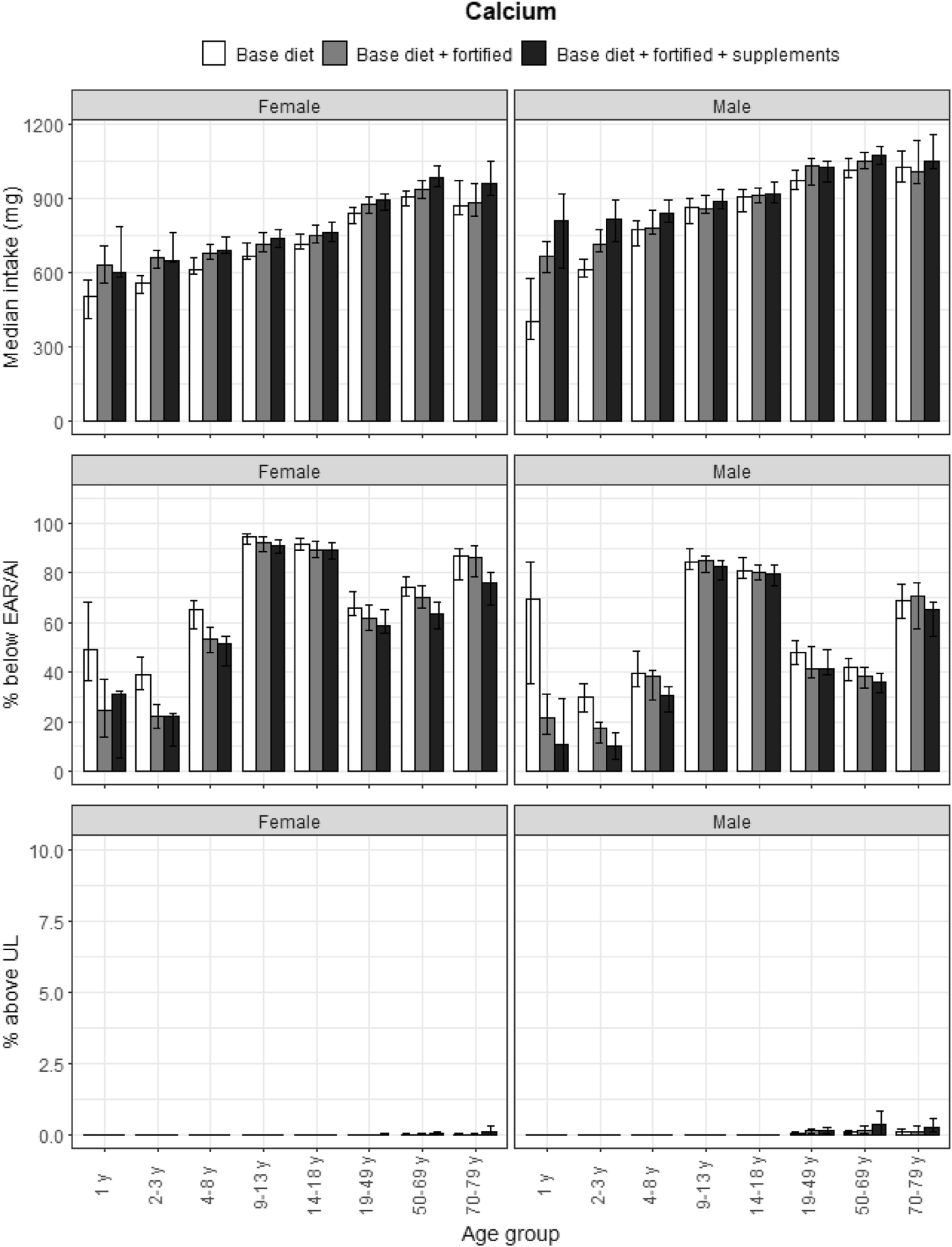
Calcium: median intakes, proportion not meeting the EAR/AI and above the UL. Median calcium intakes, proportion not meeting the EAR/AI and proportion above the UL according to age and gender categories and based on base diet, base diet with fortified foods, and total intake (base diet, fortified foods, food supplements). Data from the DNFCS, 2012–2016, *N* = 4313

Habitual Intakes of calcium followed the general trends described in the previous section and the results are presented in Fig. 1. Less than 50% of the population aged over 9 years met calcium intake requirements. More than 75% of adolescent and elderly participants did not reach the EAR; these age groups have higher intake recommendations. Intakes from fortified foods and supplements were low and did not support the Dutch population achieving their EAR. Less than 0.5% of the population exceeded the UL for any age and gender group.

### Iron

**Fig. 2:**
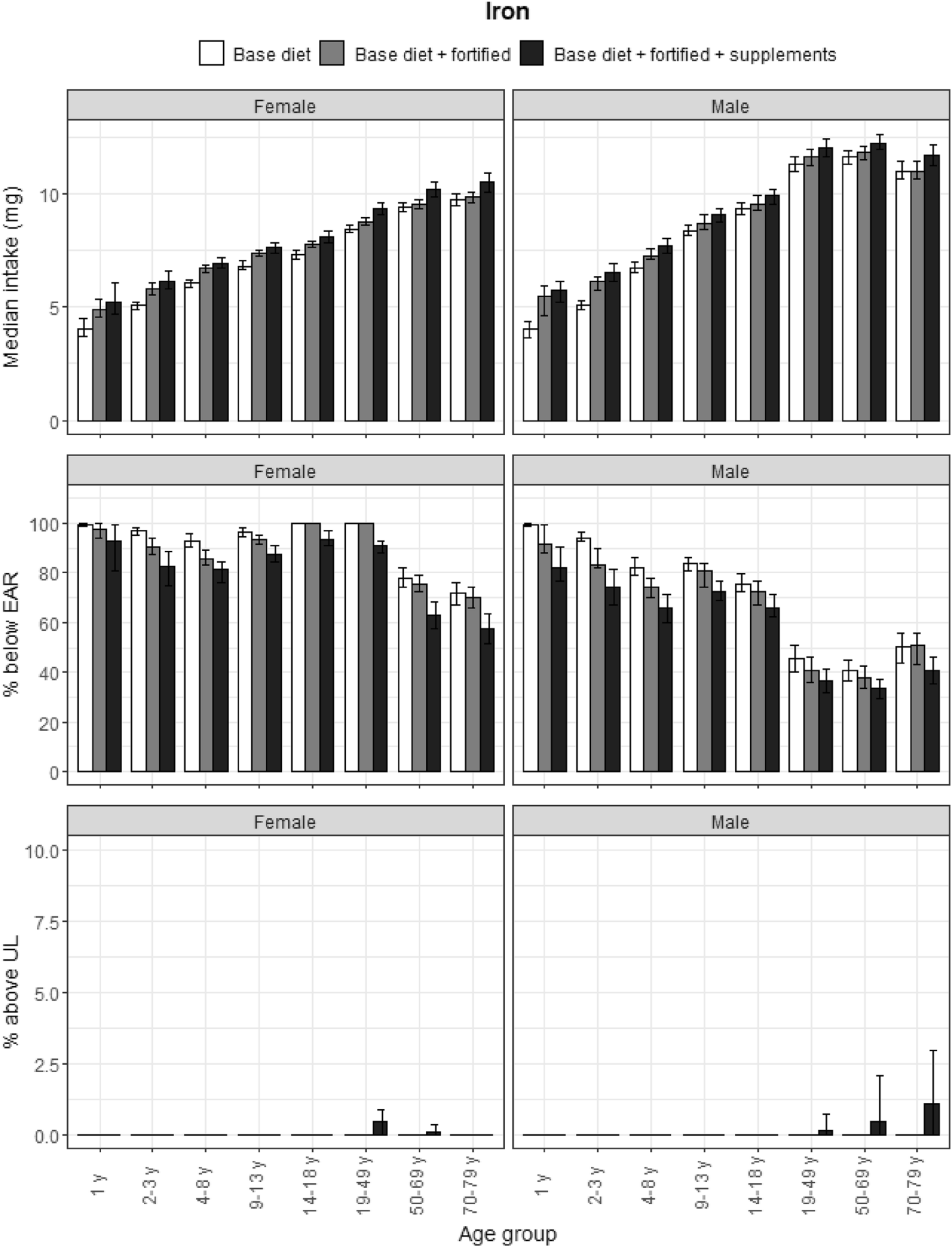
Iron: Median intakes, proportion not meeting the EAR and above the UL. Median iron intakes, proportion not meeting the EAR and proportion above the UL according to age and gender categories and based on base diet, base diet with fortified foods, and total intake (base diet, fortified foods, food supplements). Data from the DNFCS, 2012–2016, *N* = 4313

Age and gender trends described in the section above were also seen for iron (Fig. 2). Intakes were below the EAR for all female participants, and male participants aged under 19 and above 70 years. Approximately 1% of elderly men and 0.5% of women aged 19–49 years and men aged 50–69 years exceeded the UL from food supplements; non-fortified and fortified foods did not contribute to excessive intakes. Food supplements had a modest impact on reducing the proportion of participants who did not meet the EAR.

### Zinc

**Fig. 3:**
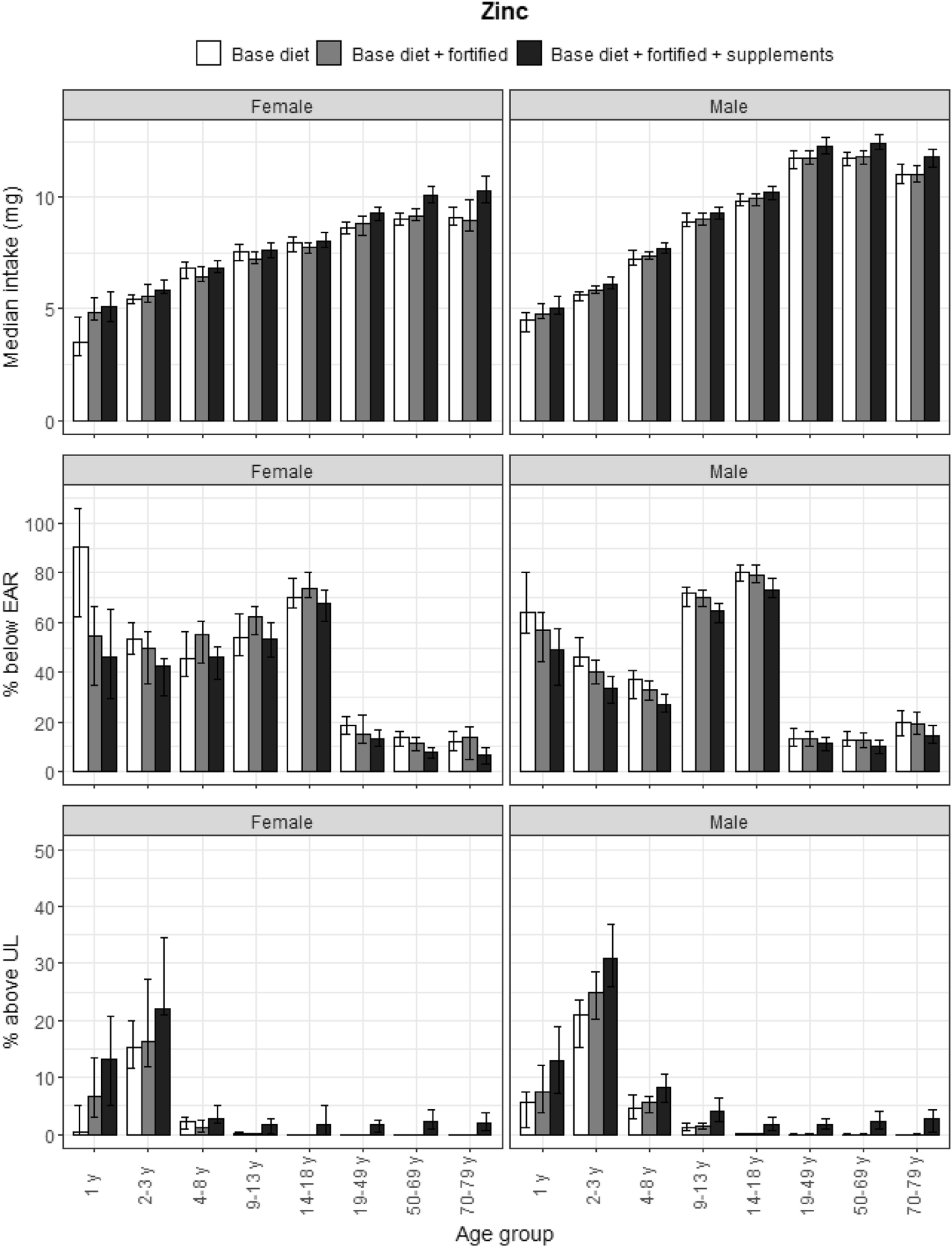
Zinc: median intakes, proportion not meeting the EAR and above the UL. Median zinc intakes, proportion not meeting the EAR and proportion above the UL according to age and gender categories and based on base diet, base diet with fortified foods, and total intake (base diet, fortified foods, food supplements). Data from the DNFCS, 2012–2016, *N* = 4313

Median zinc intakes from the base diet showed a slight decline in the adult age categories (Fig. 3). Approximately half the participants had a Habitual Intake less than the EAR for zinc. Both fortified foods and food supplements made a considerable contribution to zinc intakes in adults and reduced the proportion not meeting the EAR for this age group. The Habitual Intake of zinc from the base diet, fortified foods and food supplements all contributed to intakes above the UL. The UL was exceeded by more than 10% of toddlers, preschoolers, and school-aged children.

### Vitamin A

**Fig. 4:**
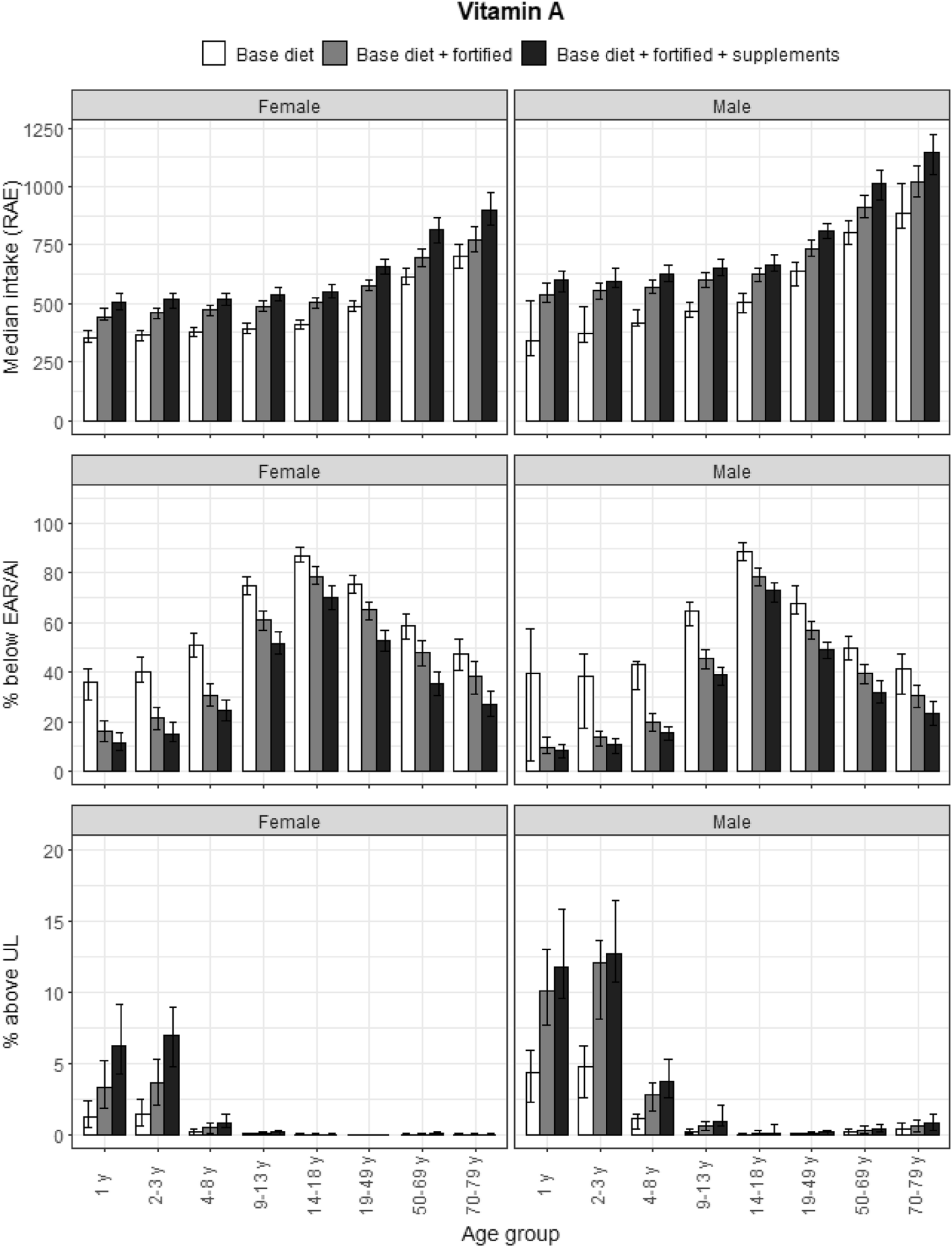
Vitamin A (RAE): median intakes, proportion not meeting the EAR and above the UL. Median vitamin A (RAE) intakes, proportion not meeting the EAR and proportion above the UL according to age and gender categories and based on base diet, base diet with fortified foods, and total intake (base diet, fortified foods, food supplements). Proportion above the UL uses preformed vitamin A (retinol and retinyl ester) intake data. Data from the DNFCS, 2012–2016, *N* = 4313

Unlike the flattening of intakes in the older adult categories, vitamin A intakes were higher for the older age categories, and particularly adults aged 70–79 years had the highest median intakes for both genders. The results are presented in Fig. 4. More than 50% of participants aged between 9 and 49 years did not meet the EAR for both genders. Despite a modest effect on median intakes, vitamin A-fortified foods and food supplements reduced the prevalence of participants not meeting the EAR for most age and gender groups. Approximately 10% of male toddlers and preschoolers, and 5% of female toddlers and preschoolers had a vitamin A intake from preformed vitamin A that exceeded the UL; the base diet, fortified foods and supplements all contributed to these high intakes.

### Vitamin B6

**Fig. 5:**
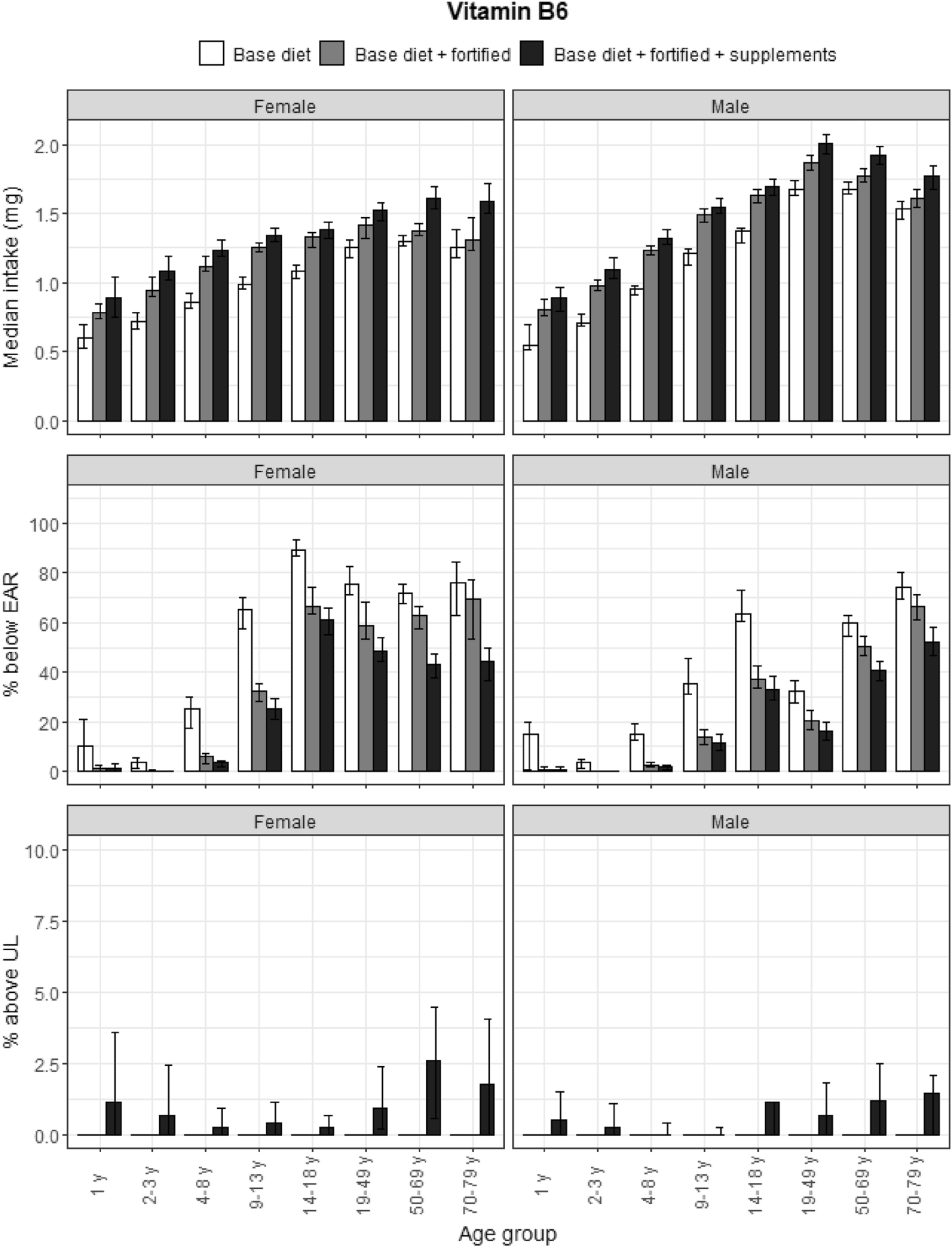
Vitamin B6: median intakes, proportion not meeting the EAR and above the UL. Median vitamin B6 intakes, proportion not meeting the EAR and proportion above the UL according to age and gender categories and based on base diet, base diet with fortified foods, and total intake (base diet, fortified foods, food supplements). Data from the DNFCS, 2012–2016, *N* = 4313

Fortified foods tended to increase vitamin B6 intakes in younger age groups, while food supplements made a stronger contribution in older adults (Fig. 5). More than 50% of girls and boys aged 14–18 years, and senior and elderly men and women, did not meet the EAR for vitamin B6, except if they consumed vitamin B6-fortified foods and food supplements. The error bars for ULs for all age groups were large. 1% or more participants in the female toddler and adult age categories, and in boys aged 14–18 and adult men exceeded the UL with food supplements.

### Folate

**Fig. 6:**
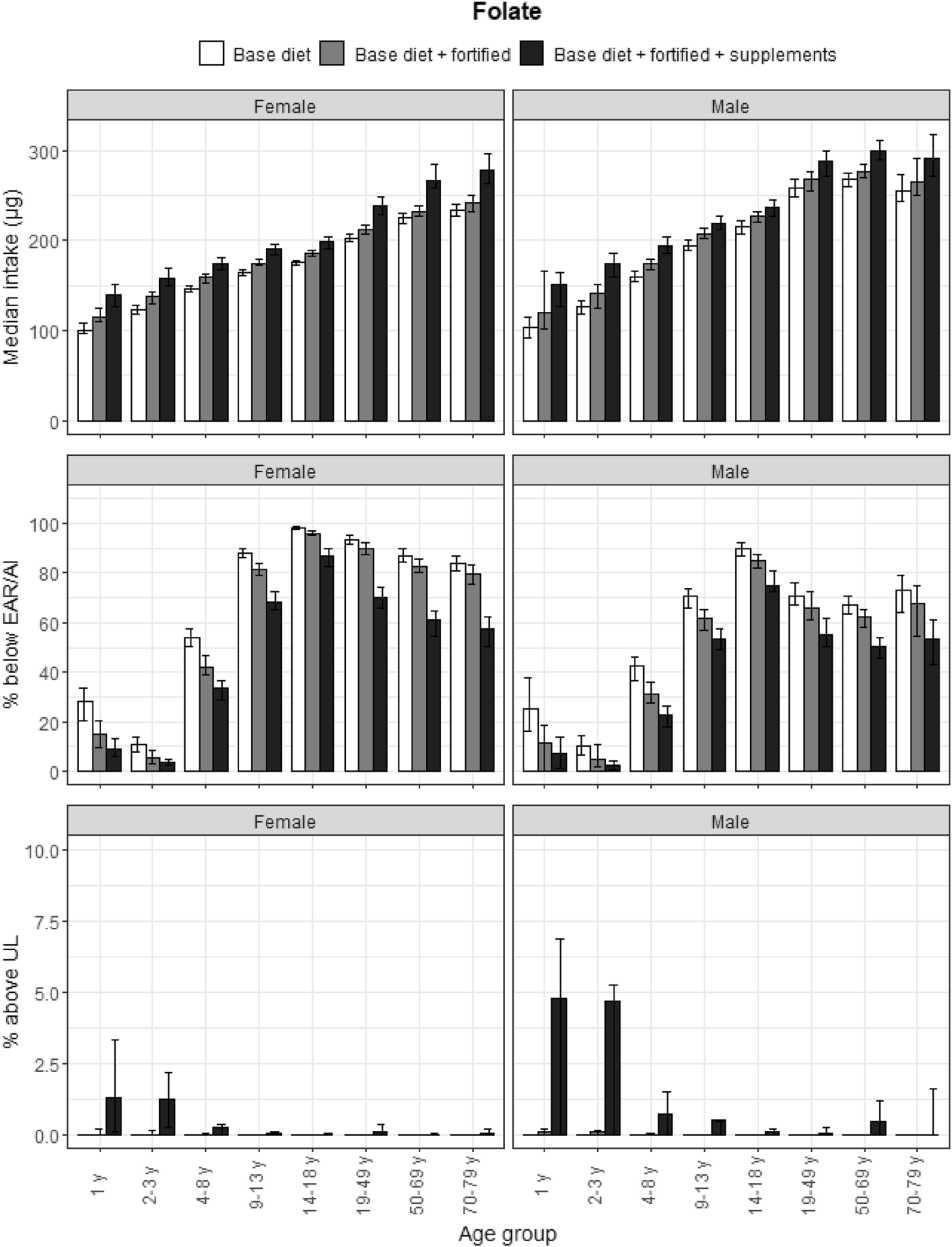
Folate equivalents: median intakes, proportion not meeting the EAR and above the UL. Median Dietary Folate Equivalent (DFE) intakes, proportion not meeting the EAR/AI and proportion above the UL according to age and gender categories and based on base diet, base diet with fortified foods, and total intake (base diet, fortified foods, food supplements). Proportion above the UL was calculated with synthetic folic acid data. Data from the DNFCS, 2012–2016, *N* = 4313

More than 50% of participants aged over 4 years did not meet the EAR for total dietary folate equivalents (folate and folic acid) from all dietary sources; this was greater than 75% for participants aged over 9 years from base and fortified diets (Fig. 6). Approximately 1% of female toddlers and preschoolers, and 4% of male toddlers and preschoolers had a folic acid intake that exceeded the UL from food supplements. Food supplements helped more than 10% of adults meet intake requirements.

### Vitamin D

**Fig. 7:**
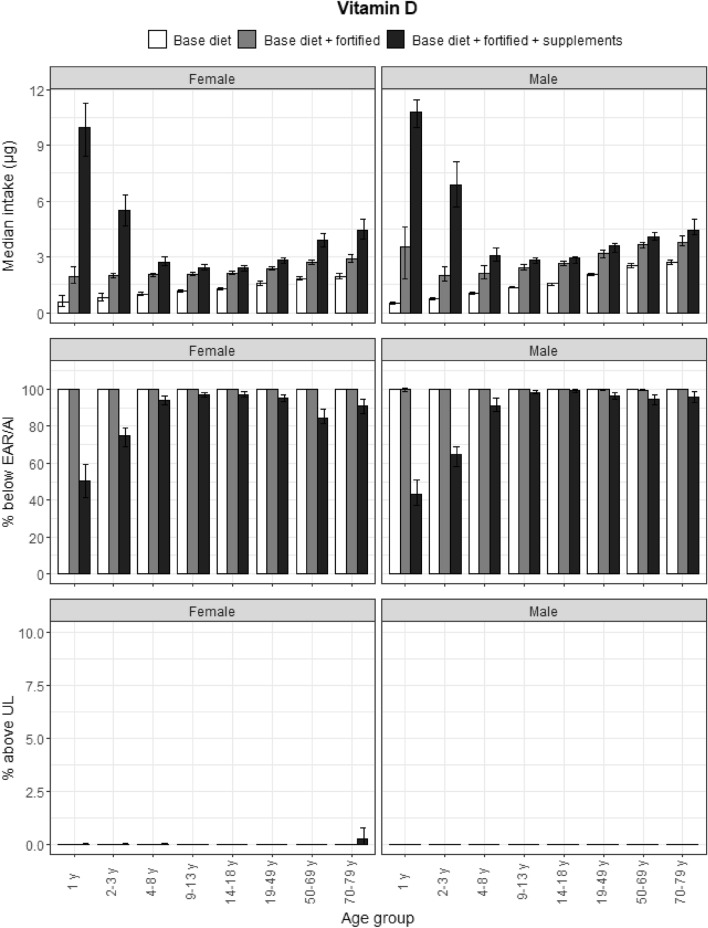
Vitamin D: median intakes, proportion not meeting the EAR/AI and above the UL. Median vitamin D intakes, proportion not meeting the EAR/AI and proportion above the UL according to age and gender categories and based on base diet, base diet with fortified foods, and total intake (base diet, fortified foods, food supplements). Data from the DNFCS, 2012–2016, *N* = 4313

There was a U-shaped relationship between age and intake for vitamin D, with median Habitual Intakes highest in the youngest and oldest age categories (Fig. 7). Both fortified foods and food supplements made a strong contribution to intakes. However, mainly food supplements but not fortified foods reduced the proportion of people not meeting the AI. Almost all participants (92%) did not meet the AI for vitamin D from base and fortified diets. Food supplements increased the proportion of toddlers and preschoolers that met the AI to 50% and 30%, respectively, but barely contributed to reduced prevalence of inadequacy for the other age and gender groups. Approximately 0.2% of elderly women exceeded the UL from food supplements.

### Vitamin E

**Fig. 8:**
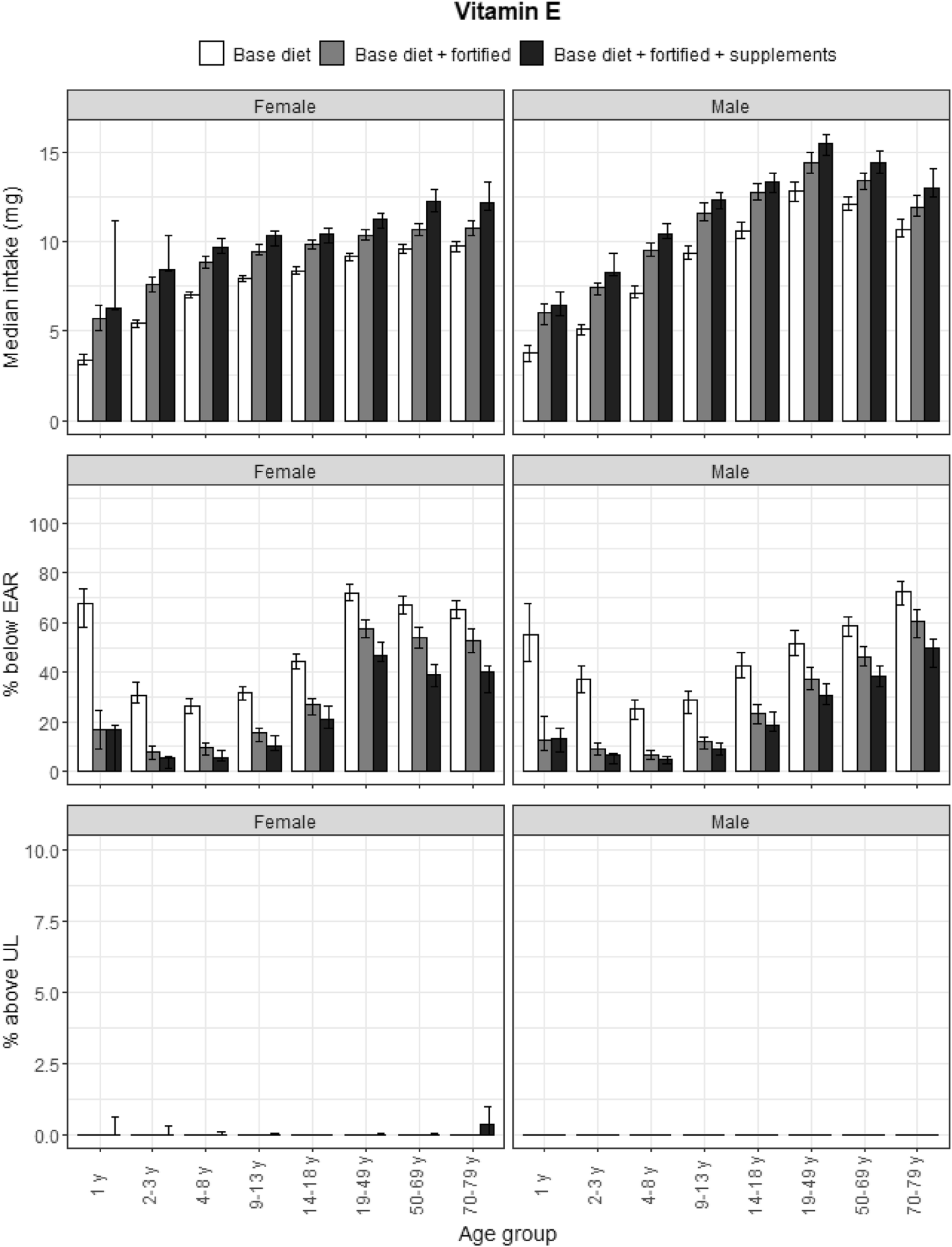
Vitamin E: median intakes, proportion not meeting the EAR/AI and above the UL. Median vitamin E intakes (mg α-Tocopherol Equivalents), proportion below the EAR/AI and proportion above the UL according to age and gender categories and based on base diet, base diet with fortified foods, and total intake (base diet, fortified foods, food supplements). Data from the DNFCS, 2012–2016, *N* = 4313

The EAR for vitamin E was not met by about 60% of female participants and 50% of male participants on a base diet (Fig. 8). For vitamin E, fortified foods more so than food supplements made a strong contribution to mean intakes in younger participants, while food supplements increased intakes in older participants. Both fortified foods and food supplements reduced the proportion of participants who had Habitual Intakes below the EAR. Less than 0.3% of elderly women exceeded the UL from food supplements.

## Discussion

This analysis identifies several nutrients in the Dutch diet with a potential for inadequate or excessive intakes for various age and gender groups, from the base diet, fortified foods and food supplements. In general, vitamin D intakes were inadequate for almost all Dutch people, and sub-groups had inadequate intakes of calcium, iron, zinc, vitamin A, folate, vitamin E, particularly adolescents and adult women, and from people who did not consumer fortified foods or food supplements. Toddlers and preschoolers had intakes that were more likely to be adequate; fortified foods and supplements appear to make an important contribution to adequacy in this age group. More than 1% of certain sub-groups exceeded the UL for folic acid, vitamin B6, preformed vitamin A, zinc and iron, particularly for toddlers and preschoolers. When assessing the intake of vitamins and minerals in any population, it is important to consider both inadequate and excessive intakes for population health.

These results are in line with other reports of nutrient intake in the Netherlands and other European countries [24–26]. In the Netherlands, the main report based on the survey also found low intakes for calcium, iron, vitamin A, vitamin B6 and folate [11], and identified adolescent girls and women to be at risk of inadequate intakes. The results reported by de Jong and colleagues broadly agree with ours, although different age categories limit direct comparisons, and intakes from food supplements were not included [6]. A smaller cross sectional study conducted in 2012–2013 in 254 Dutch elderly subjects found similar proportions not meeting the EAR for vitamins B6 and D, although calcium intake shortfalls were not identified [27]. The article from Flynn and co-workers similarly looked at intakes from the total intake and reported median intakes comparable to ours [3]. However, the P95 in the Flynn article occurred at a lower intake, indicating a narrower distribution. Despite a similar methodology used in terms of calculating Usual Intakes from two 24-h dietary recalls, the data in the Flynn analysis was collected between 1997 and 2006 in several surveys and perhaps reflects changes in nutrient intakes over time. In addition, the broader age categories used in the Flynn analysis might attenuate the effect of fortified foods and food supplements in more narrowly defined age groups. The analysis conducted by Flynn et al. also did not include data from children aged under 4 years [3]. A comparison of four countries in Europe found 23% of adults in France and Denmark, 34% of adults in Italy and 62% of adults in the Czech Republic did not meet vitamin A intakes [25]. Vitamin D intakes were inadequate for most (> 92%) people; nevertheless, sun exposure in the summer months in the Netherlands may contribute to some extent to maintaining an adequate vitamin D status in winter; in 2014, 26.5% of adults were below deficient (< 50 nmol/L) and 66.6% were below optimal (75 nmol/L) [28]. Fortified foods and food supplements made a moderate contribution to reducing inadequate intakes in children aged 3 years and younger, and in women aged 50 + years.

More than 50% of the entire population did not meet the EAR for calcium, iron and folate from the total intake. While there are no nationally representative surveillance studies for biochemical markers of these nutrients in the Netherlands, small surveys give an indication about whether these dietary inadequacies are potentially contributing to deficiency status. A survey of 400 Dutch children aged 6 months–3 years found iron deficiency in 19% [29]. Iron deficiency was also seen in 9% of 376 Dutch and migrant elderly [30] and 4% of healthy adults, with a higher prevalence of iron deficiency in women, in a study of 348 users of a digital lifestyle program [31]. Thus, the large proportion of inadequate intakes in toddlers is likely to contribute to iron deficiency in toddlers. For the other age groups, the low prevalence of deficiency despite low intakes of iron may mean that factors affecting the bioavailability and absorbance of iron mediate the correlation between intake and status. On the other hand, there is uncertainty in translating the results of several small cross sectional studies to population iron status.

Although there is a lack of data on folate status in the Netherlands, in a study of 348 healthy adults, folate deficiency was found in 14.1% [31]. No established biomarkers exist for calcium, therefore the impact of low calcium intakes can only be tracked through the prevalence of diseases related to calcium intakes, such as osteoporosis. Osteoporosis causes considerable morbidity and associated health care costs in the Netherlands [32]; however, it is difficult to ascertain the effect that inadequate calcium intake has on osteoporosis occurrence and severity. These results indicate that better surveillance of nutrient biomarkers and strategies to address vitamin and mineral deficiencies and their effects on health in the Netherlands are warranted.

When comparing age and gender groups, adolescents were more likely to have inadequate calcium, zinc and vitamin A intakes, women were more likely to have a lower intake of iron, vitamins B6, folate and vitamin E, and adults also had low vitamin A intakes. Even though the elderly are often identified as being at greater nutritional risk [33], intakes were more likely to be adequate in the elderly age category for our analysis. It is possible that the relatively young cut-off at 79 years for the sample excluded older elderly participants at greatest nutritional risk. Food supplements also reduced inadequacy for this age group for vitamin B6, vitamin D and vitamin A, a finding that is in line with a cross sectional study conducted in Dutch elderly during a similar time period [27].

Our analysis found that Habitual Intakes from all sources exceeded the UL for more than 1% of the following age and gender groups: toddlers (folic acid, total vitamin A), adults (vitamin B6), all age groups and especially toddlers (zinc), and elderly men (iron). Toddler zinc intakes also exceeded the UL for non-fortified foods.

The UL for folic acid is set based on the potential masking of vitamin B12 deficiency symptoms, rather than toxicity concerns from high intakes [18]. While it is difficult to assess vitamin B12 deficiency in the Netherlands due to a lack of national surveillance data, one study has investigated vitamin B12 status in the general Dutch population. This article investigated the incidence of macrocytic anemia and low folate and vitamin B12 in 161,548 undiagnosed patients at a diagnostic center [34]. The researchers found that approximately one quarter of Dutch adults had low serum vitamin B12, with this proportion increasing to 30% in the elderly (80 years and older); macrocytic anemia was found in 1.3% of the total population and was considerably lower in patients with low serum B12 (1.9%) compared to low serum folate (15.6%). Based on these results, it is difficult to assess whether B12 deficiency masking by high folate intakes is occurring. Recent changes to the way DFEs are calculated in Europe based on additional data on folate forms may affect the proportion above the UL [35]; we were not able to incorporate this information into the analysis because the calculation of DFEs is performed within the NEVO database.

The UL for zinc is based on potential inhibition of copper absorption at high zinc intakes. The 97.5% of total zinc intakes is close to the UL in many countries, and was not considered to be a concern by EFSA [18]. In addition, although median intakes of zinc increase from approximately 5 mg/d for the youngest age group to approximately 10 mg/d for the adult age groups, the UL increases from 7 mg/d for the youngest age group to 25 mg for the adults; therefore, the margin between median intakes and the UL is much narrower for young children, leading to a greater proportion exceeding the UL.

Attention needs to be paid to preformed vitamin A intakes in young children, particularly from fortified foods and food supplements. The safety margin between the UL and actual dietary intake of high consumers (P95) is known to very small for vitamin A. National surveys in France [16] and Germany [36] show that people who consume products containing liver on a regular basis exceed the UL due to the high content of preformed vitamin A in liver. In line with other countries, our data also show that the UL was already exceeded by the base diet for consumers with a high vitamin A intake, and an additional analysis found that liver consumers were more likely to exceed the P95 (results not shown). Recently, a randomized, controlled trial conducted in Filipino toddlers found no evidence of chronic vitamin A toxicity despite chronic high intakes of preformed vitamin A, and the authors suggest that the cut-off for hypervitaminosis A in the liver is too conservative [37]. The UL for vitamin A for children was set by scaling the adult UL of 3000 μg/day, which in turn is based on a 2.5-fold lower level than the lowest-observed-adverse-effect level of 7500 μg/day for hepatotoxicity from chronic intakes [22]. These intakes involve preformed vitamin A and not intakes from pro-vitamin A carotenoids, for which no UL has been set, however intakes greater than 20 mg per day are contraindicated in smokers [22]. Replacing part of the preformed vitamin A with pro-vitamin A carotenoids such as beta-carotene in food supplements and fortified foods designed for toddlers and small children is a strategy that can reduce high intakes of preformed vitamin A while still contributing to total vitamin A intakes.

Food supplement use in the toddler age group was the highest of all age groups. It is possible that current advice from the government to supplement children under the age of 4 years with a vitamin D supplement combined with picky eating in young children means that caregivers are more likely to give a multivitamin to ensure nutritional adequacy in this age group: Our data showed that 73% of female toddlers and 74% of male toddlers received a food supplement, which was the highest of the age/gender groups (Table 1). The list in Online Material: Supplemental Table 5 shows that vitamin D was the most frequently consumed supplement in the 1–3 years age group, and chewable multivitamins for the 4–8 year age category. The data on individual food intakes showed that the use of follow-on toddler formulas contributes to intakes from fortified foods for this age group (results not shown). Approximately 20% of toddlers in this survey use follow-on infant formula: Formula users had higher intakes of vitamins B1, B2, folic acid, C, D, E, and iron and zinc, and an increase in the proportion exceeding the UL for zinc [38]. In a Spanish population, the use of fortified milk including follow-on formula improved adequacy in zinc, vitamin A and vitamin E, while the proportion of children exceeding the UL also increased for zinc and vitamin A for the youngest age group. More than 5% of toddlers in this study exceeded the UL for zinc, vitamin A and selenium regardless of the type of milk they consumed [39]. Changes to fortification and supplementation practices should consider both risk of deficiency and excessive intakes.

This analysis has several strengths. The dataset is representative for the Netherlands and the data collection methodology is robust with the use of two dietary recalls and validated data collection software. The Habitual Intake analysis corrects for within-person variance and thus improves estimations of population intakes below or above a cut-off, particularly in the tails of the distribution, which is important for estimates of the UL in particular. We were also able to individually model the intake distribution according to age, using intakes from fortified foods, and including food supplement intake, which provides a comprehensive analysis of overall nutrient intakes in sub-groups that vary considerably in their normal diet.

A potential weakness of the analysis is the subject selection method via a consumer panel. This could potentially lead to bias and a non-representative sample: Participants who volunteer for the panel may differ in their knowledge of health and nutrition compared those who decline to participate [40]. Thus, dietary intakes obtained from this survey may show a healthier pattern than the general population. A further weakness is our calculation of the base (i.e., non-fortified) diet. In some cases, such as vitamin A fortification of margarines, this approach was accurate because the other ingredients in margarine do not contribute to vitamin A intakes. On the other hand, for other fortified foods such as iron in legume-based vegetarian burgers, the intrinsic iron content contributes to the total iron content of the food. The exact amount of intrinsic and fortified iron in vegetarian burgers depends on the recipe used by the manufacturer, which was not provided in the dataset. Thus, the subtraction method we used will potentially result in lower base diet intakes than actually are consumed, and also a greater difference between base and base and fortified diets in terms of absolute intake and the contribution toward meeting nutrient intake requirements. In addition, incorrect reporting of food intakes, particularly under-reporting, is a “fundamental obstacle” in nutritional surveys [41], and likely affects estimations of food and thus micronutrient intake in the DNFCS. While the magnitude of this effect is difficult to quantify, we could assume that intakes are higher than reported. It is thus likely that the actual proportion not meeting the EAR/AI is lower and the proportion exceeding the UL is higher than what we found in our analysis due to under-reporting.

Our analysis applies to data from the Netherlands, thus the patterns of dietary intakes by food type or age/gender group seen might not be seen in countries that share a similar geography or degree of economic development. Recent national nutrient intake data are generally lacking within the EU countries. Data are even more scarce for nutrient intakes obtained from base foods, food supplements and fortified foods.

Future research should combine dietary survey intake data together with blood sample analysis for nutritional status to assess whether nutrient intakes reflect biomarkers of dietary exposure. This is important because nutritional status of several vitamins is not only dependent on food intake but on other factors such as nutrient bioavailability, digestion, absorption, synthesis by the microbiota, and cutaneous synthesis from UV light for vitamin D. As fortified foods had only a modest impact on overall intakes, the use of fortification to improve micronutrient intakes to reduce the consequences of deficiencies could be explored further [42]. In addition, our analysis identified a number of population sub-groups at risk of micronutrient deficiencies or excessive intakes of certain micronutrients: potentially, targeted nutrition education interventions and the involvement of community dietitians should be developed to mitigate these risks.

## Conclusions

Representative dietary intake data from the Netherlands revealed that a large part of the population has inadequate intakes of calcium, iron, zinc, vitamin A, vitamin D, vitamin E and folate. Population sub-groups especially at risk of inadequate intakes were adolescents and women. Fortified foods and food supplements made a modest contribution to intakes and nutrient adequacy. Toddlers were most likely to exceed the UL, which occurred for zinc, preformed vitamin A and folic acid, particularly for food supplement users, but also for the base diet for zinc and vitamin A. A careful appraisal of the effects of deficiency and excess of these nutrients on population health is warranted. Future nutritional surveys should consider combining biological (blood/plasma) markers of nutritional status with dietary intake analysis. These results can be used to inform policy makers and to design nutritional interventions to improve micronutrient intakes in the Netherlands.

### Supplementary Information

Below is the link to the electronic supplementary material.

Supplementary file1 (PDF 72 KB)

Supplementary file2 (XLSX 59 KB)

## Data Availability

Public and private organizations may use the DNFCS data for research purposes, education, or teaching. Access to and permission to use the data may be granted by sending a motivated application to the RIVM. The form can be obtained via the following website: https://www.rivm.nl/en/dutch-national-food-consumption-survey/data-on-request (accessed 2nd August 2023).

## Declarations

### Standards of reporting

A STROBE checklist has been provided with this submission for reporting of observational studies.
