# Supplementary material for: Micronutrient intakes in the Dutch diet: foods, fortified foods and supplements in a cross sectional study
Source: Eur J Nutr. 2023 Aug 5;62(8):3161–79. doi: 10.1007/s00394-023-03219-4 (PMC10611853; doi:10.1007/s00394-023-03219-4)
Supplement: Supplementary file 1 — Supplementary file1 (PDF 72 KB) [file 394_2023_3219_MOESM1_ESM.pdf]

Supplemental tables for: Micronutrient Intakes in the Dutch Diet: Foods, Fortified Foods and Supplements in a Cross-Sectional Study

European Journal of Nutrition

Authors: Julia K. Bird[1] 0000-0001-6015-3576, Maaike J. Bruins[2*] 0000-0001-9517-4617, Marco Turini[2]

[1]Bird Scientific Writing, Wassenaar, The Netherlands

[2]DSM Nutritional Products, Kaiseraugst, Switzerland

[*]Corresponding author: Maaike J. Bruins, maaike.bruins@dsm.com

Supplemental Table 1: Dietary Estimated Average Requirements (EAR) or Adequate Intakes (AI) by age and gender category

	Vitamin A	Vitamin B6	Folate	Vitamin D	Vitamin E	Calcium	Iron	Zinc
	µg RAE[1]	mg	µg DFE[2]	µg	mg αTE[3]	mg	mg	mg
	EAR	EAR/AI	EAR/AI	AI	EAR/AI	EAR/AI	EAR	EAR
1 to 3 y M+F	231	0.3	57	10	4.5	395	4	5
4 to 8 y M+F	289	1	100	10	5.5	553	4	5
9 to 13 y M	461	1	150	10	8	947	6	8
9 to 13 y F	463	1	150	10	7	868	4	7
14 to 18 y M	692	1	200	10	10	868	6	9
14 to 18 y F	540	1	200	10	8	868	6	7
19+ y M	615	1.1	200	10	13	750	6	6.4
19+ y F	525	1.1	200	10	11	750	6.5	5.7
Conversion Factor M	1.3	1.4	1.5			1.3	1.8	1.4
Conversion Factor F	1.3	1.4	1.5			1.3	2.5	1.2
Conversion Factor M+F	1.3	1.4	1.5			1.3	2.2	1.3

[1] RAE: Retinol Activity Equivalents [2] DFE: Dietary Folate Equivalent, [3] α-TE: α-Tocopherol Equivalents. For adults 19 years and above the AR and AI values for vitamins and minerals as set by the Dutch Health Council in 2018 were used.[i] For all the other age groups the (temporary) nutrition reference values from 2014 were used.[ii] Because AR values were only available for adults and not children, they were estimated from the adult Population Reference Values (PRV) by dividing by the calculated adult 2 x STDEV for males, females, or both genders (ranging from 1.2-1.4 except iron: 1.8-2.1), as reported in the table.

Supplementary Table 2: Tolerable Upper Intake Levels (UL) for adults

Vitamin A[1]	Vitamin B6	Folic acid[2]	Vitamin D	Vitamin E	Calcium	Iron	Zinc
µg RAE[3]	mg	µg DFE[4]	µg	mg αTE[5]	mg	mg	mg
The Netherlands / EFSA[6]				*EFSA*[7]			
3000	25	1000	100	300	2500	No UL set	25

[1] From preformed retinol and retinyl esters. [2] From synthetic folic acid. [3] RAE: Retinol Activity Equivalents [4] DFE: Dietary Folate Equivalent, [5] α-TE: α-Tocopherol Equivalents. [6] The UL set by the Dutch Health Council[iii] and EFSA were the same. [7] Where no UL by the Dutch Health Council was available, the UL set by EFSA[iv] was selected.

Supplemental Table 3: Description of dataset

	Subgroup	Overall	Taking a dietary supplement (questionnaire)		p-value
			Yes	No	
N	Total	4,313	2,084	2,229	-
Sex N (%)	Male	2,165 (50.2)	915 (43.9)	1,250 (56.1)	<0.001
	Female	2,148 (49.8)	1,169 (56.1)	979 (43.9)	
Age (years) (mean (SD))	Total	28.61 (25.24)	25.74 (25.47)	31.30 (24.72)	<0.001
Age category N (%)	Boys, 1-3 years	332 (7.7)	245 (11.8)	87 (3.9)	<0.001
	Girls, 1-3 years	340 (7.9)	259 (12.4)	81 (3.6)	
	Boys, 4-8 years	261 (6.1)	137 (6.6)	124 (5.6)	
	Girls, 4-8 years	259 (6.0)	148 (7.1)	111 (5.0)	
	Boys, 9-13 years	259 (6.0)	101 (4.8)	158 (7.1)	
	Girls, 9-13 years	260 (6.0)	118 (5.7)	142 (6.4)	
	Boys, 14-18 years	270 (6.3)	81 (3.9)	189 (8.5)	
	Girls, 14-18 years	254 (5.9)	98 (4.7)	156 (7.0)	
	Men, 19-30 years	260 (6.0)	91 (4.4)	169 (7.6)	
	Women, 19-30 years	256 (5.9)	118 (5.7)	138 (6.2)	
	Men, 31-50 years	259 (6.0)	86 (4.1)	173 (7.8)	
	Women, 31-50 years	264 (6.1)	142 (6.8)	122 (5.5)	
	Men, 51-70 years	264 (6.1)	80 (3.8)	184 (8.3)	
	Women, 51-70 years	258 (6.0)	150 (7.2)	108 (4.8)	
	Men, 71-79 years	260 (6.0)	94 (4.5)	166 (7.4)	
	Women, 71-79 years	257 (6.0)	136 (6.5)	121 (5.4)	
Season of first dietary recall N (%)	Spring	1022 (23.7)	503 (24.1)	519 (23.3)	0.015
	Summer	992 (23.0)	435 (20.9)	557 (25.0)	
	Autumn	1031 (23.9)	515 (24.7)	516 (23.1)	
	Winter	1268 (29.4)	631 (30.3)	637 (28.6)	

Unweighted counts/percentages

Supplemental Table 4: Dietary Supplement Category Use

Dietary supplement category	Category	In winter (N (%)*)	Outside winter (N (%)*)
Participant used any dietary supplements (vitamins and/or minerals or other)	Yes	1997 (95.8)	1536 (73.7)
	No	87 (4.2)	548 (26.3)
Frequency of use of dietary supplements: Multivitamins without minerals	Never	1752 (87.7)	1391 (90.6)
	<1 day per month	19 (1.0)	17 (1.1)
	1-3 days per month	22 (1.1)	14 (0.9)
	1 day per week	13 (0.7)	6 (0.4)
	2-3 days per week	44 (2.2)	21 (1.4)
	4-5 days per week	39 (2.0)	17 (1.1)
	6-7 days per week	108 (5.4)	70 (4.6)
Frequency of use of dietary supplement: Multivitamins with minerals	Never	931 (46.6)	766 (49.9)
	<1 day per month	28 (1.4)	26 (1.7)
	1-3 days per month	58 (2.9)	51 (3.3)
	1 day per week	55 (2.8)	47 (3.1)
	2-3 days per week	131 (6.6)	112 (7.3)
	4-5 days per week	191 (9.6)	95 (6.2)
	6-7 days per week	603 (30.2)	439 (28.6)
Frequency of use of dietary supplement: β-carotene	Never	1953 (97.8)	1503 (97.9)
	<1 day per month	6 (0.3)	8 (0.5)
	1-3 days per month	4 (0.2)	3 (0.2)
	1 day per week	3 (0.2)	3 (0.2)
	2-3 days per week	4 (0.2)	1 (0.1)
	4-5 days per week	6 (0.3)	4 (0.3)
	6-7 days per week	21 (1.1)	14 (0.9)
Frequency of use of dietary supplement: Vitamin B complex	Never	1790 (89.6)	1399 (91.1)
	<1 day per month	23 (1.2)	17 (1.1)
	1-3 days per month	18 (0.9)	6 (0.4)
	1 day per week	17 (0.9)	7 (0.5)
	2-3 days per week	16 (0.8)	9 (0.6)
	4-5 days per week	23 (1.2)	11 (0.7)
	6-7 days per week	110 (5.5)	87 (5.7)
Frequency of use of dietary supplement: Folic acid	Never	1941 (97.2)	1483 (96.5)
	<1 day per month	8 (0.4)	5 (0.3)
	1-3 days per month	2 (0.1)	2 (0.1)
	1 day per week	3 (0.2)	3 (0.2)
	2-3 days per week	6 (0.3)	4 (0.3)
	4-5 days per week	3 (0.2)	3 (0.2)
	6-7 days per week	34 (1.7)	36 (2.3)
Frequency of use of dietary supplement: Vitamin C	Never	1475 (73.9)	1207 (78.6)
	<1 day per month	41 (2.1)	32 (2.1)
	1-3 days per month	63 (3.2)	50 (3.3)
	1 day per week	56 (2.8)	31 (2.0)
	2-3 days per week	76 (3.8)	34 (2.2)
	4-5 days per week	62 (3.1)	27 (1.8)

	6-7 days per week	224 (11.2)	155 (10.1)
Frequency of use of dietary supplement: Vitamin D	Never	1220 (61.1)	913 (59.4)
	<1 day per month	18 (0.9)	15 (1.0)
	1-3 days per month	33 (1.7)	21 (1.4)
	1 day per week	32 (1.6)	24 (1.6)
	2-3 days per week	60 (3.0)	43 (2.8)
	4-5 days per week	74 (3.7)	61 (4.0)
	6-7 days per week	560 (28.0)	459 (29.9)
Frequency of use of dietary supplement: Calcium / vitamin D combined	Never	1819 (91.1)	1408 (91.7)
	<1 day per month	12 (0.6)	9 (0.6)
	1-3 days per month	16 (0.8)	7 (0.5)
	1 day per week	5 (0.3)	7 (0.5)
	2-3 days per week	15 (0.8)	14 (0.9)
	4-5 days per week	13 (0.7)	6 (0.4)
	6-7 days per week	117 (5.9)	85 (5.5)
Frequency of use of dietary supplement: Vitamin E	Never	1898 (95.0)	1449 (94.3)
	<1 day per month	11 (0.6)	10 (0.7)
	1-3 days per month	11 (0.6)	6 (0.4)
	1 day per week	4 (0.2)	9 (0.6)
	2-3 days per week	5 (0.3)	6 (0.4)
	4-5 days per week	12 (0.6)	10 (0.7)
	6-7 days per week	56 (2.8)	46 (3.0)
Frequency of use of dietary supplement: Calcium	Never	1874 (93.8)	1447 (94.2)
	Less than one day per month	7 (0.4)	7 (0.5)
	1-3 days per month	13 (0.7)	4 (0.3)
	1 day per week	5 (0.3)	4 (0.3)
	2-3 days per week	10 (0.5)	4 (0.3)
	4-5 days per week	9 (0.5)	13 (0.8)
	6-7 days per week	79 (4.0)	57 (3.7)
Frequency of use of dietary supplement: Iron / Iron pill	Never	1911 (95.7)	1471 (95.8)
	Less than one day per month	7 (0.4)	8 (0.5)
	1-3 days per month	20 (1.0)	11 (0.7)
	1 day per week	9 (0.5)	4 (0.3)
	2-3 days per week	9 (0.5)	7 (0.5)
	4-5 days per week	8 (0.4)	7 (0.5)
	6-7 days per week	33 (1.7)	28 (1.8)

*Unweighted counts/percentages

Supplemental Table 5: Top three dietary supplement types by age category

Age category	Supplement name	Percentage*	Age category	Supplement name	Percentage*
Boys 1-3 y	Vitamin D drops for children water basis	10.37	Girls 1-3 y	Vitamin D drops for children (water basis)	9.46
Boys 1-3 y	Vitamin D - water suspension	8.74	Girls 1-3 y	Vitamin D drops	7.24
Boys 1-3 y	Vitamin D unspecified	7.11	Girls 1-3 y	vitamin D - water suspension	6.84
Boys 4-8 y	Junior 3+ chewable vitamins - Multifruit	12.03	Girls 4-8 y	Junior 3+ chewable vitamins - Multifruit	14.47
Boys 4-8 y	Child Multi vitamins and minerals (flavored)	6.33	Girls 4-8 y	Child Multi vitamins and minerals (flavored)	11.95
Boys 4-8 y	Vitamin D	6.33	Girls 4-8 y	Junior 3+ chewvitamins	6.92
Boys 9-13 y	Junior 3+ chew vitamins - Multifruit	7.92	Girls 9-13 y	Vitamin C 70 mg	6.72
Boys 9-13 y	Child Multi	6.93	Girls 9-13 y	Child Multi vitamins and minerals (flavored)	5.88
Boys 9-13 y	Fish oil for children	5.94	Girls 9-13 y	Junior 3+ chewable vitamins - Multifruit	4.20
Boys 9-13 y	Multi AZ	5.94	Girls 9-13 y	Multi A-Z complete	4.20
Boys 14-18 y	Multi A-Z complete	8.82	Girls 9-13 y	Multi Boost 12+	4.20
Boys 14-18 y	Vitamin C 70 mg	5.88	Girls 14-18 y	Multi A-Z complete	5.88
Boys 14-18 y	Multi all in 1 chew tablet	4.41	Girls 14-18 y	Magnesium	4.90
Men 19-30 y	Multi A-Z complete	9.92	Girls 14-18 y	Cranberry	3.92
Men 19-30 y	Complete Chew vitamins - strawberry	3.31	Girls 14-18 y	Vitamin D - tablets	3.92
Men 19-30 y	Vitamin C 1000 powerful	3.31	Women 19-30 y	Multi A-Z complete	11.58
Men 19-30 y	Vitamin C 70 mg	3.31	Women 19-30 y	Multi All in 1	4.21
Men 31-50 y	Multi A-Z complete	9.85	Women 19-30 y	Total 30	4.21
Men 31-50 y	Total 30	4.55	Women 31-50 y	Multi A-Z complete	7.01
Men 31-50 y	Complex Forte (tablet)	3.79	Women 31-50 y	Not specified	2.58
Men 51-70 y	Glucosamine pure	4.23	Women 31-50 y	Total 30	2.58
Men 51-70 y	Multi A-Z 50+ Vital	4.23	Women 51-70 y	Vitamin D	4.74
Men 51-70 y	Vitamin C-1000 time released	3.17	Women 51-70 y	Multi A-Z 50+ Vital	3.39
Men 71-79 y	Total 30	4.40	Women 51-70 y	Vitamin D 10 mcg	2.93
Men 71-79 y	Multi A-Z complete	2.52	Women 71-79 y	Vitamin D	3.81
Men 71-79 y	Multi Vital 65+ Extra immunity with Q10 and lutein	2.52	Women 71-79 y	Multi A-Z complete	3.46
*Percentage of total per age/gender group			Women 71-79 y	Glucosamine pure	2.60

Supplemental Table 6: Most commonly consumed fortified foods in DNFCS 2012-2016

Fortified food name	# of times consumed in DNFCS[1]	Vitamin/mineral[2]								
		Ca	Fe	Folate	Zn	A	B6	D	E	Other[3]
Half-fat margarine	7291	0	0	0	0	1	0	1	0	0
Margarine	3783	0	0	0	0	1	0	1	0	0
Bake-and-fry fat	2504	0	0	0	0	1	0	1	1	0
Fruit cordial	1558	0	0	0	0	0	0	0	0	1
Mixed fruit drink (not 100% juice)	1005	0	0	0	0	0	0	0	0	1
Single fruit drink (not 100% juice)	762	0	0	0	0	0	0	0	0	1
Breakfast pap	364	0	0	0	0	0	0	0	0	1
Infant formula	297	1	1	1	1	1	1	0	1	0
Fat, not specified	254	0	0	0	0	1	0	1	1	0
Rosehip cordial	253	0	0	0	0	0	0	0	0	1
Drink brand Taksi/Djoezz and similar	227	0	0	0	0	0	0	0	0	1
Soy milk	226	1	0	0	0	0	0	0	0	0
Cornflakes	194	0	1	1	0	0	1	0	0	0
Caffeinated energy drink	191	0	0	0	0	0	1	0	0	0
Fruit-based cordial	170	0	0	0	0	0	0	0	0	1
Cordial, type not specified	160	0	0	0	0	0	1	0	1	0
Fruit and grain bar	117	1	1	0	0	0	1	0	0	0
Cookies with milk-based topping	107	1	1	0	0	0	1	0	0	0
Children’s breakfast pap	101	1	1	0	0	1	1	0	1	0
Pure juice	96	0	0	0	0	1	0	1	0	0
Enriched white bread	90	0	1	0	1	0	1	0	0	0
Rosehip cordial, special	83	0	0	0	0	0	0	0	0	1
Rosehip and fruit cordial, diluted	81	0	0	0	0	0	0	0	0	1
Baby/toddler cookie	73	1	0	0	0	0	0	0	0	0
Soy yoghurt	70	1	0	0	0	0	0	0	0	0
Vitamin-enriched water	67	1	0	0	0	0	1	0	0	0
Breakfast cereal rice crispies	64	0	1	1	0	0	1	0	0	0
Child dairy dessert	60	1	0	0	0	0	0	0	0	0
Breakfast cereal loops/circles	53	1	1	0	0	0	1	0	0	0
Children’s pap, ready to eat	52	1	1	1	1	1	1	0	1	0
Enriched brown bread	48	0	1	0	1	0	1	0	0	0
Meal replacement milkshake	42	1	1	1	1	1	1	0	1	0
Spread	42	0	0	0	0	1	0	1	0	0
Sport energy drink	42	0	0	0	0	0	0	0	0	1
Cookie	40	1	0	0	0	0	0	0	0	0
Rosehip and fruit cordial	39	0	0	0	0	0	0	0	0	1
Mixed fruit juice (100% juice)	39	0	0	0	0	0	0	0	0	1
Mixed fruit drink (not 100% juice)	38	0	0	0	0	0	0	0	0	1
Rosehip cordial, type not specified	37	0	0	0	0	0	1	0	1	0

[1]248,883 individual food and drink items were consumed in total in the DNFCS. [2]Fortified foods flagged with “1” contain the vitamin or mineral specified. [3]Other micronutrients could be vitamins B1, B2, B3, B12, C, K, or minerals copper, iodine, phosphorus, potassium, selenium.
